# A machine-learning scraping tool for data fusion in the analysis of sentiments about pandemics for supporting business decisions with human-centric AI explanations

**DOI:** 10.7717/peerj-cs.713

**Published:** 2021-09-17

**Authors:** Swarn Avinash Kumar, Moustafa M. Nasralla, Iván García-Magariño, Harsh Kumar

**Affiliations:** 1 IIIT Allahabad, Uttar Pradesh, India; 2 Department of Communications and Networks Engineering, Prince Sultan University, Riyadh, Saudi Arabia; 3 Universidad Complutense de Madrid, Madrid, Spain; 4 Instituto de Tecnología del Conocimiento, UCM, Madrid, Spain; 5 Peoples’ Friendship University of Russia, Moscow, Russia

**Keywords:** Machine learning, Pandemics, COVID-19, Sentiment analysis, Decision support system, Business intelligence

## Abstract

The COVID-19 pandemic is changing daily routines for many citizens with a high impact on the economy in some sectors. Small-medium enterprises of some sectors need to be aware of both the pandemic evolution and the corresponding sentiments of customers in order to figure out which are the best commercialization techniques. This article proposes an expert system based on the combination of machine learning and sentiment analysis in order to support business decisions with data fusion through web scraping. The system uses human-centric artificial intelligence for automatically generating explanations. The expert system feeds from online content from different sources using a scraping module. It allows users to interact with the expert system providing feedback, and the system uses this feedback to improve its recommendations with supervised learning.

## Introduction

The applications of sentiments analysis has been widely applied for dealing with COVID-19 pandemic (Alamoodi et al., 2020). These applications analyze the sentiments for different outbreak incidents using social medial. This information has been considered valuable for mitigating the pandemic with several approaches for making citizens aware of the situation for accepting social restrictions, obtaining successful results. However, the existence of different sources requires data fusion for handling all the data together.

Although this approach is designed for pandemics in general, it has only been tested during COVID-19 pandemic. Thus, it may only be applied in pandemics with similar features to COVID-19. In particular, the two key aspects are probably the mortality in the different age ranges and the ways of transmission. The former aspect can influence on the perceived risk of the different population groups considering ages, and consequently in the economic activities related to these age groups. The latter aspect about ways of transmission may influence on the restrictions imposed by governments and consequently on the different related economic sectors.

The impact of COVID-19 on economics has been outrageous in most countries, as highlighted for example for India and developing countries (Singh & Misra, 2020), in the first half year of the pandemics. However, economics are evolving and adapting for the circumstances of pandemics, conforming a new emerging economics field concerning global COVID-19 pandemic (Zhang & Ramse, 2020), which is now being researched and taugh in prestigious institutions such as Shanghai Business School and George Fox University.

Machine learning (ML) has been widely applied for sentiment analysis with different approaches (Wang et al., 2021), including supervised ML, gradual ML, using many different techniques such as support vector machines (SVM), random forest (RF), multilayer perceptrons (MLPs) and deep neural networks (DNN). In particular, the keys are which document features to use and how to reduce the manual labeling effort when facing a new domain or context.

Although sentiment analysis has shown its utility in business decision-making such as investment strategies (Petit, Lafuente & Vieites, 2019) and gaining business insight through Twitter analysis (Reyes-Menendez, Saura & Filipe, 2020), the pandemic outbreaks are deeply changing the business models and ecosystems (Anker, 2021). Some businesses are adapting to the new circumstances, while others are closing down. The literature still lacks appropriate methodologies and studies on how to apply sentiment analysis in the context of pandemics, to discover new opportunities related with the real-time circumstances of pandemic and the associated restrictions.

The assessment of business strategies usually measures performance as profits and number of sales to determine which products or services are more successful. This is already well studied and analyzed in previous works such as (Mokhtar & Wan-Ismail, 2012). However, this continuously changing scenario of pandemics may not rely only in past history as the circumstances are rapidly changing. This article aligns with the line of works that argue that sentiment analysis can bring some light in business decisions for anticipating which products and services may be more successful in new circumstances (Oppong et al., 2019).

Human-centered artificial intelligence (HAI) supports the automatic generation of explanations for the decisions suggested by ML. ML techniques such as deep learning (Gao, Wang & Shen, 2020c) usually provide classification results without explanations about the reasons behind such classifications. However, among others, Bryson & Theodorou (2019) stated that the generation of explanations of HAI is necessary for the long-term stability of society, for reaching wise decisions based on proper supervision of the automatically suggested decisions based on the underlying reasons. For instance, the explainable MLP provides estimated reasons of each decision based on the analysis of learned neuron weights (García-Magariño, Muttukrishnan & Lloret, 2019).

In this line of research, this work proposes a novel approach for applying sentiment analysis using data fusion for reinforcing and empowering companies in pandemics situations so that economy can improve in this context with HAI. This approach can be useful especially for small and medium enterprises (SMEs) that are looking for new strategies to survive.

The remainder of this paper is organized as follows. Next section introduces the most relevant related work, highlighting the gap of the literature that is covered by the current work. ‘ML Scraping Tool for Monitoring Sentiments about Pan-demics’ presents the proposed approach based on a scraping tool with supervised ML for identifying business opportunities based on sentiment analysis on combinations of certain business decisions and pandemic circumstances. ‘Experimentation’ describes the experimentation of this approach illustrated with two different case studies respectively about a bar business and an information technology (IT) business. Finally, ‘Conclusion and Future Work’ mentions the conclusions and the future research lines.

## Related work

Some sentiment analysis tools use ML. For instance, the system of Sharma & Sharma (2020) performed sentiment analysis on Twitter to predict public emotions with ML. Their goal was to identify behavioral attributes of individuals based on their activity in social media.

Sentiment analysis has been used for many different applications. One of its main applications is the analysis of the citizens’ sentiments about political aspects. For example, Kinyua et al. (2021) used sentiment analysis and ML for analyzing the impact of president Trump’s tweets.

Several works apply ML for sentiment analysis. In these works, normally ML requires input from documents labeled with sentiments. As Wang et al. (2021) recently indicated, one of the key challenges is to reduce the initial effort of manually labeling the initial *corpus* with emotions. More concretely, they focused on the aspect-level sentiment analysis. They applied gradual ML in which easy instances are automatically inferred, based on an estimated certainty. Hard cases were manually labeled in small stages. Their results were competitive in comparison with DNN approaches.

In the fields of ML and sentiment analysis, most works use public resources. For instance, Twitter messages are commonly used, as one can observe in the work by Soumya & Pramod (2020). They applied the common ML techniques of SVM, random forest (RF) and naive Bayes. They used document features considering bag of words, terms frequency, inverse document frequency, Unigram with Sentiwordnet and negation of words. In addition, Ghiassi & Lee (2018) also used Twitter information for showing their approach with transferable lexicon in supervised ML for achieving high accuracy in sentiment analysis considering specific domains. The feature selection usually depended on the domain and its selection was challenging in general. They extracted a small Twitter specific lexicon set, and showed that the usage of this set was useful for sentiment analysis in Twitter regardless of specific domains.

Sentiment analysis is well-known for its capacity in supporting business decisions. For instance, analysis of investors’ sentiments can be useful in supporting business decisions based on the need of investments on certain decisions related with specific products. More concretely, Petit, Lafuente & Vieites (2019) applied principal component analysis (PCA) on web searches to shape profiles of investors and their sentiments towards certain aspects. As further described by Reyes-Menendez, Saura & Filipe (2020), exploratory sentiment analysis supports marketing challenges gaining business insights. They used the tweets from hashtag “#MeToo” and used supervised learning with SVM. They found the importance of gender equality and inclusiveness in marketing.

Business activity is one of the major concerns in COVID-19 pandemic. First, Europe is planning the post-COVID agenda for the research in business and management (Anker, 2021). Business ecosystems have suffered deep changes as one can observe in the underlying market circumstances and the boundary conditions between business and society. The consumer values have changed to a new era of selflessness. Supply chains splitting have accelerated with pandemics, conforming new supply mechanisms and structures. The constant possibility of disruption as new normal has changed both the way of consuming and the business strategies. Cloud computing has showed to be effective to solve problems related to COVID-19 (Singh et al., 2021) such as the needs raised by people working on their homes communicating and collaborating online. This has been especially useful in the healthcare domain. This implies the need of cloud datacenters with the corresponding needs of energy, which can be addressed with renewable energy sources (Gao, Wang & Shen, 2020b). In this context, ML has been applied for the prediction of workload in cloud computing (Gao, Wang & Shen, 2020a). Although computing power needs of COVID-19 are being properly handled in the literature, the literature misses to provide decision support systems for assisting small local companies in addressing the new challenges of adapting their services and products. Another relevant aspect is how to nominate the new concepts related to COVID-19 in order to apply natural language processing over the text related to COVID-19. In this context, Alag (2020) proposed a COVID-19 ontology related to clinical trials. This aligns with the existing literature about ontology frameworks (Gheisari et al., 2021), in which the used ontology is key in properly analyzing texts. Nevertheless, none of these works about ontologies have been used in the context of business strategies for supporting companies in adapting their strategies in pandemics.

Several works highlight the role of sentiments during the pandemic. For instance, Buckman et al. (2020) indicated that COVID-19 pandemic was causing disruptions in economic activity, stating their proposed Daily News Sentiment Index was a useful real-time indicator. They compared their index with survey-based consumer sentiment. In the same line, Nemes & Kiss (2021) used sentiment analysis over social media on COVID-19 circumstances. They applied natural language processing techniques combined with a recurrent neural network for sentiment classification. Although these works support that automatic sentiment analysis can bring real-time useful information in COVID-19 changing scenarios like in our work, these works did not provide a methodology for exploring and selecting service/products to sell based on this information, as our current work does.

HAI is argued to be necessary in scenarios related to pandemics, including the customers’ preferences in medical affairs and ethics in the transformed society in COVID-19. More concretely, in the medical affairs, Bedenkov et al. (2021) explicitly argued the need of HAI for extracting useful information of data sets conformed of conversations with customers. HAI could provide useful information with proper explanations regarding certain aspects. However, they did not provide any specific implemented system for the identified needs. In the field of ethics, Wilthagen et al. (2020) discussed the advantages and drawbacks of ML in delicate decisions, and the possibility of unperceived discrimination cases if not using proper HAI. However, this work did not propose an actual application of HAI for supporting business growth in pandemics circumstances, as the current work does. Furthermore, HAI has been applied for supporting the decisions of policy makers and stakeholders concerning food security with simulated scenarios considering different conditions (How, Chan & Cheah, 2020). Nevertheless, this work neither considered sentiments, pandemics nor private businesses when designing their decision-support system with HAI. Therefore, none of these works developed a system for supporting business decisions with HAI in the context of pandemics.

In summary, ML has been widely used for sentiment analysis. Most of these works explore sentiments based on social media and other online resources, being Twitter one of the most common resources. Domain has been proved to be essential in the selection of the document features. Sentiment analysis has prove n to be crucial in business, and business organizations and strategies are deeply changing with the current COVID-19 pandemic. Nevertheless, the literature lacks of the works that actually address the incorporation of sentiment analysis in business strategies specifically designed to take advantages of the new business opportunities in the COVID-19 pandemic context, for achieving the common goal of business survival in the current pandemic, especially in SMEs. The current work covers this gap of the literature, by proposing a ML scraping tool for monitoring sentiments of the real-time pandemic circumstances to support business decisions with explanations generated with HAI.

## Ml scraping tool for monitoring sentiments about pandemics

In this approach, we propose a scraping tool for analysing online content about pandemic from different sources for implementing a decision support system (DSS) that helped businessmen and entrepreneurs in deciding how to initiate new businesses or maintain existing ones.

**Figure 1:**
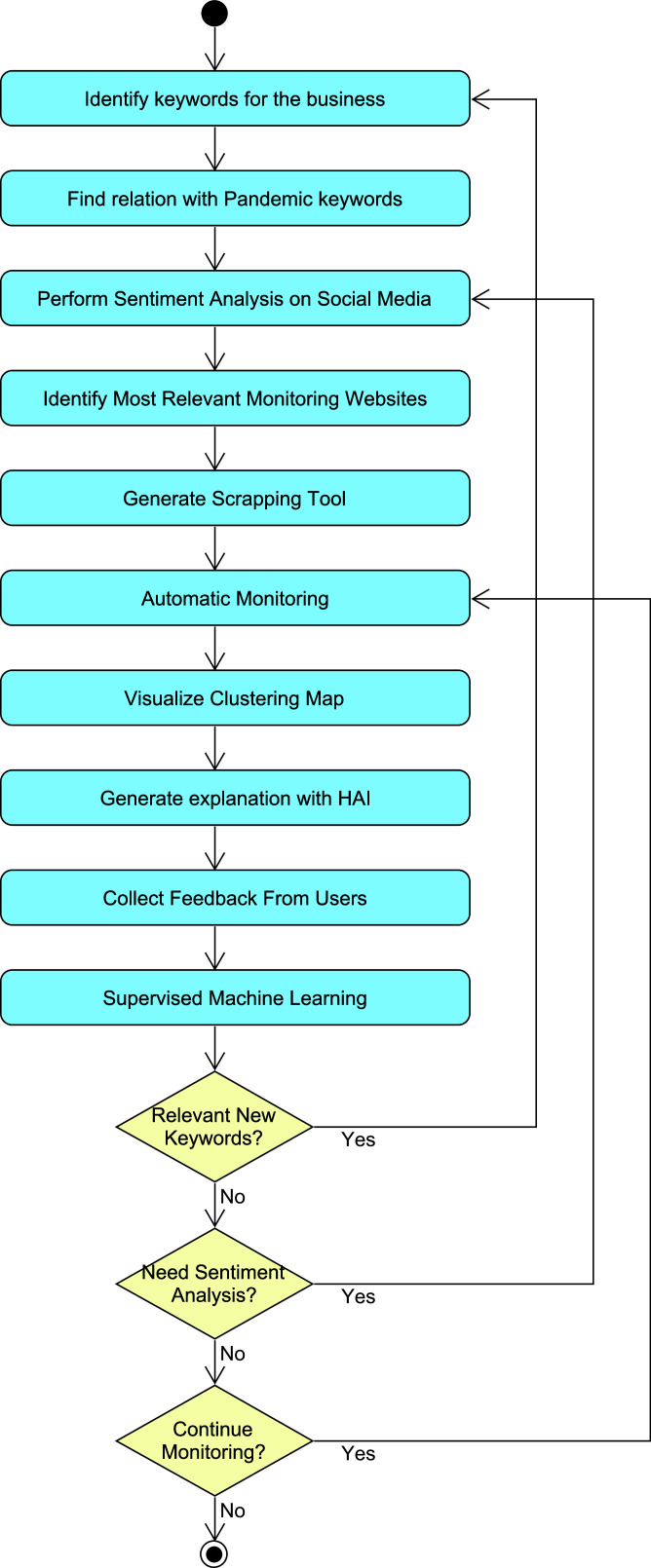
Block diagram of the scraping tool.

Figure 1 presents the block diagram of the proposed approach for generating and using the scraping tool that is used to support decisions. The first step was to identify the keywords of the business, which depended on its specific domain. The application asked each user to enter either these keywords or a paragraph indicating the most relevant aspects. In the latter option, the tool extracted a set of relevant words.

The next step was to identify the words from the pandemics that were most related with the selected keywords. For this purpose, the tool departed from a pool of words related to pandemics, and performed an online analysis of which words were most related in several web pages about news. For this purpose, the tool use d the Selenium library for searching on different websites which pairs of words (formed by one business keyword and one pandemic keyword) are most frequent.

**Figure 2:**
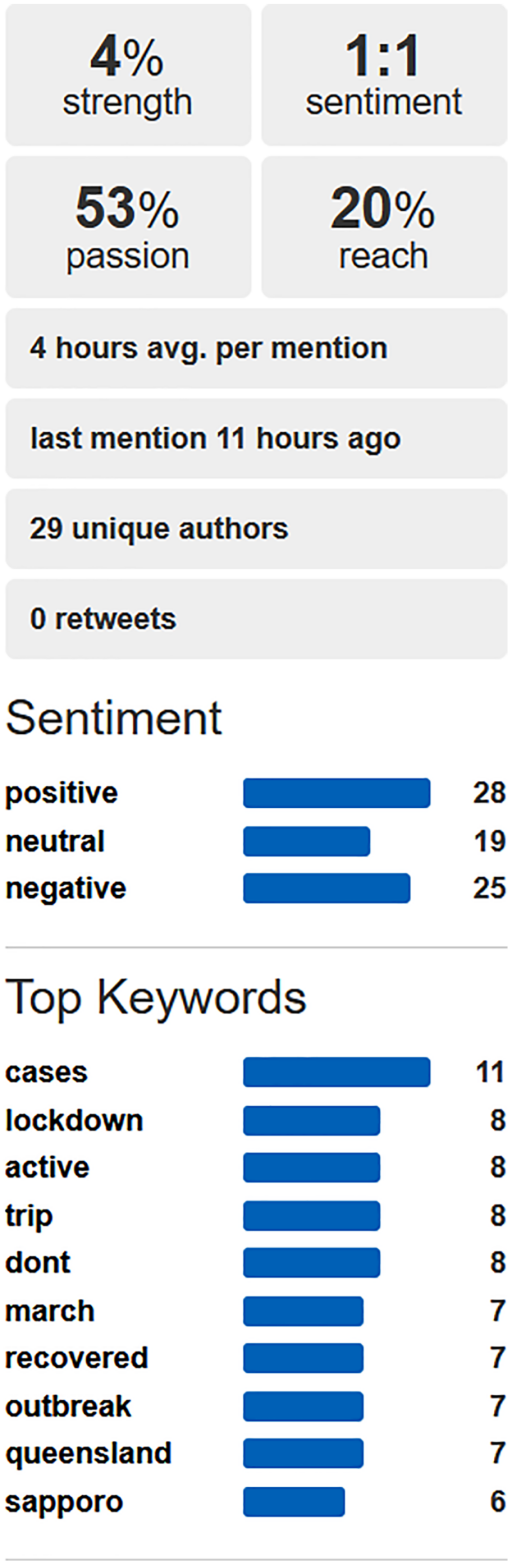
Sentiment analysis in social media about a pair of keywords related respectively with pandemic and a business.

After this, the tool performed a sentiment analysis on social media to determine the sentiments about the pandemic keywords most related with the business alone or in combination with the business keywords. This information was useful information for taking decisions, and the relevant outputs were presented to the user. The tool fed from data provided by the Social Mention website (http://socialmention.com). Figure 2 shows an example of the social information used for a bar business, and the information extracted for the particular combination of “restriction” in the context of pandemic and “beer” as a word related to the business.

After this, the proposed tool identified the most relevant links in the social media related with the relevant combinations of words, considering as well how dynamic the websites were. Once this identification was performed, the tool generate d a scraping tool to collect dynamic information from each relevant website. It detected in which HTML environment these words were mentioned, taking tags, attributes and parent tags into account among others. In occasions, the generated scraping tool needed to be manually improved by an experienced programmer in order to obtain more relevant results.

The automatic monitoring module periodically used the scraping tool in order to obtain relevant information that could help businessmen in taking decisions such us when launching offers or alter their prices. It also provide d information about company strategies that could have a positive impact in the sentiment of clients, and how evolution of the pandemic could affect their companies.

**Figure 3:**
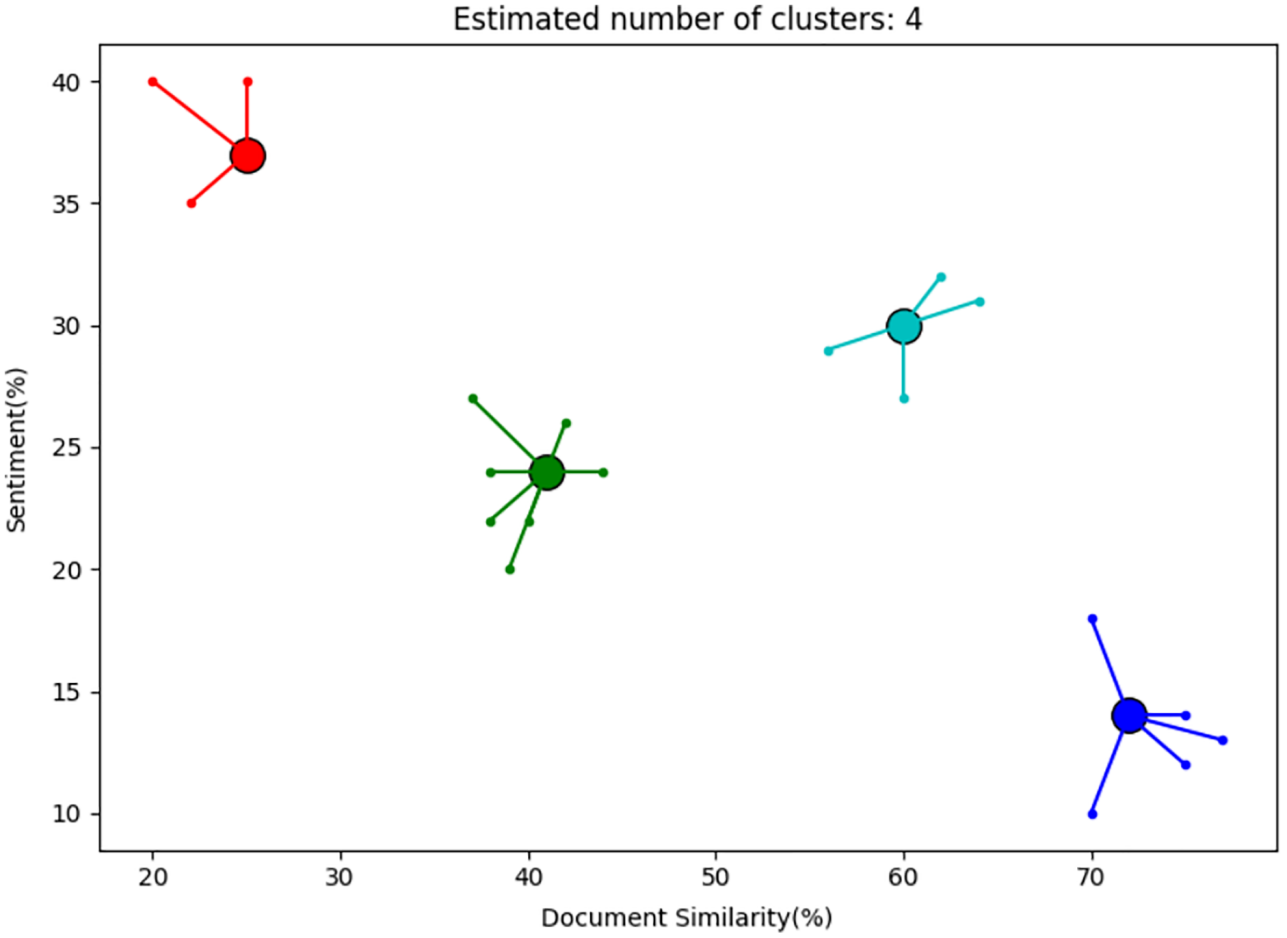
Clustering map.

The automatic monitoring tool provided a clustering map and a recommendation automatically generated with HAI. Figure 3 shows an example of the clustering map provided by this tool. In the clustering map, each point represents a document with certain similarity for a given set of keywords (x-axis) and the sentiment represented as a percentage (y-axis). In this particular example, the documents represented news about bars and COVID-19 restrictions. The clustering identified clusters of documents with similar features, so the user was able to analyze the cluster of documents that was the most relevant. The user was instructed to select clusters with high document similarity, since these may be the most relevant to their business (the bar in this case). The user was told that both positive and negative sentiments could be useful for either emphasize or avoid certain products or services. In this map, the tool used the affinity propagation clustering algorithm with the implementation provided by the Scikit-learn library (Pedregosa et al., 2011). In each combination, the clustering map considered the three dimensions for each document, which were (a) the similarity with the specific business keywords, (b) the similarity percentage with the specific pandemic keywords, and (c) the sentiment analysis of the given document. Alternatively, the two former dimensions were also calculated as one unique similarity considering both business and pandemic keywords. The sentiment measurement was calculated with the following equation:

(1) $$sentiment = 100*\displaystyle{{positive + K*neutral} \over {positive + neutral + negative}}$$

where “positive”, “negative” and “neutral” are the number of documents parts retrieved for a given combination of keywords with respectively positive, negative and neutral sentiments. K is a constant in the interval [0, 1], and its recommended default value is 0.5, as neutral sentiment is considered to be in the middle between positive and negative sentiments. In this work, all the experimentation used this default value, but other researchers may tune this value in specific business contexts.

The tool collected feedback from users for performing a supervised ML approach in which this feedback was used either to start resetting/adding keywords or to repeat the whole process. It could repeat the sentiment analysis if new information about sentiments was provided by the user. Otherwise, it continue d the monitoring until user s decide d to stop it.

In order to implement the ML, the proposed approach used the term frequency and inverse document frequency considering customized sets of keywords, as document features. The relevance of documents indicated by users’ feedback was used in the supervised learning. We used Scikit-learn library with Python programming language for using the ML techniques of multinomial naive Bayes (NB) classifier, MLPs, k-nearest neighbors (KNN), SVMs and multinomial Bayesian.

In order to automatically generate an explanations, we used two techniques, The first one analyzed the weights of MLP using our approach of explainable MLPs (García-Magariño, Muttukrishnan & Lloret, 2019). We also tested a HAI approach on KNN, based on generating an explanation based on the nearest neighbor document. An example of an automatically generated explanation follows:

*In the learned model for business opportunities in pandemics, the most relevant input combination for estimating that you have negative emotions about vaccines is that you were thinking about AstraZeneca and clots*.

The proposed approach is iterative and needed three bifurcations (as one can see in the block diagram) to iterate going back to different steps depending on the new input data. Firstly, if the user provides new keywords, then the process needs to be repeated from the beginning since the identification of keywords is the first step. In some occasions, the pandemic circumstances and related restrictions change and periodically some iterations need to consider again the sentiments as these may change, going back to the middle of the process. Finally, if not any of the previous circumstances occur, the selected websites can be monitored in short and frequent iterations.

## Experimentation

The experimentation was conducted in two different scenarios respectively related with a bar and a information technology company and introduced in the next two subsections.

### Bar business

A bar wanted to take some decisions related on how to proceed to keep profitable their business. In the COVID-19 pandemic, many restriction were applied such as the mandatory closing time in Madrid (Spain). After some brainstorming, the owners though of the options of focusing their promotion efforts and marketing on (a) breakfasts (b) lunches, (c) snacks, (d) delivery to homes, (e) low-cost/cheap services, and (g) low-cost/cheap tickets for post-pandemic services.

In order to support their decisions, we followed the proposed approach as indicated for the following steps of the proposed approach:

- Identify keywords for the business: The keywords respectively associated with each possible business decision were respectively (a) breakfasts (b) lunches, (c) snacks, (d) delivery to homes, (e) bear, (f) low-cost/cheap services, (g) special offers, and (h) low-cost/cheap tickets for post-pandemic services. In addition, the common keywords in all the decisions were bar, restaurant, tapas, terraces and Madrid.
- Find relation with pandemic keywords: In most decisions, we found relation with the following keywords: pandemic, Covid19, restriction, 10 pm, 22 h, Madrid, commercial activity and closing time.
- Perform sentiment analysis on social media: For each possible decision, we gathered the set of keywords of this business decision with the Pandemic keywords, and used brief and non-redundant set of keywords, for performing analysis on the Social Mention website. On this analysis, the business owners identified that many French people were coming to Madrid to enjoy bars because of the more relaxed restrictions compared with France. The business owner found this information useful and translated all their offers to French in order to catch the attention of all these potential French customers.
- Identify most relevant monitoring websites: The two most relevant websites were *ruptly.tv* and *foxnews.com*, which respectively provided information about French consumers and generous customers that were willing to prepay food and drinks to keep tapas bars open in pandemic. In this bar scenario, web scraping started from Social Mention website, and this provided a list of relevant websites related with the selected keywords. The bar owner was instructed to check the first websites in the ranking, and selected the ones that he found most appropriate.
- Generate scraping tool: The proposed tool was generated for monitoring these two websites considering the most relevant sets of works for the two most promising lines of business: (1) potential French customers with keywords French, Madrid, tourists, terraces, escape, COVID-19 restrictions, and (2) prepay offers with the keywords bar, prepay, food, drink, tapas, open, coronavirus, pandemic.
- Automatic monitoring: These scraping tool was used to monitor these two websites for 2 weeks. The automatic monitoring tool was designed to check the relative frequency of “French” term in the news related with “Pandemics” and “Restrictions”, and updated a list of the most relevant news considering “French” in this context in the two selected websites. This monitoring tool notified the user whenever some new news came up in this list.
- Visualize clustering map: The map was monitored to compare different offers for respectively French customers and prepay offers.
- Generate explanation with HAI: An example of generated explanation was “*In the learned model for business opportunities in pandemics, the most relevant input combination for estimating that you have negative emotions about bars is that you were thinking about Covid and Tracking*”. Thus, the bar owner thought about incorporating a tracking system for checking temperature and avoid people with COVID-19 compatible symptoms in the bar.
- Collect feedback from users: The owners found especially useful the finding about potential French customers, as the cost of translation to French was very low in comparison to the potential earnings from French customers.
- Supervised learning: In the supervised learning, the users selected the two most relevant news, which were “Spain: French tourists fill Madrid terraces to escape COVID-19 restrictions” (Ruptly, 2021) and “Hundreds of bar customers prepay for food and drinks to keep tapas joint open amid coronavirus pandemic” (Leggate, 2020). The supervised learning use d these documents to train the system for presenting first the documents most similar to these with supervised positive feedback.

### IT business

In IT business, the context was more advantageous than in the previous case study, since people used more technology for communication in hard restrictions about pandemics such as confinement. However, the technology usage had changed. We applied the following steps proposed by our approach for supporting IT business decisions

**Figure 4:**
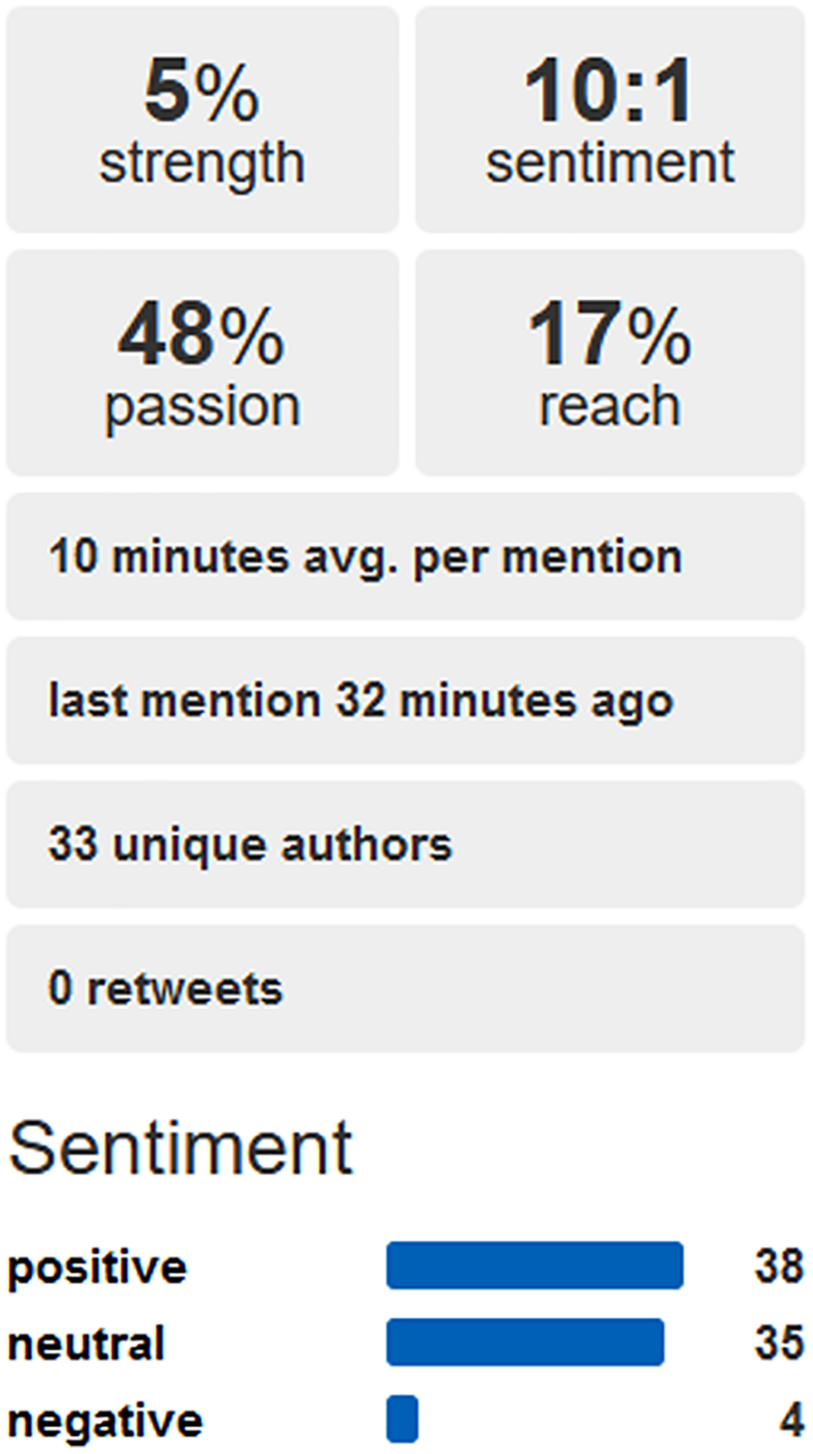
Sentiment analysis in social media about AI and pandemic.

**Figure 5:**
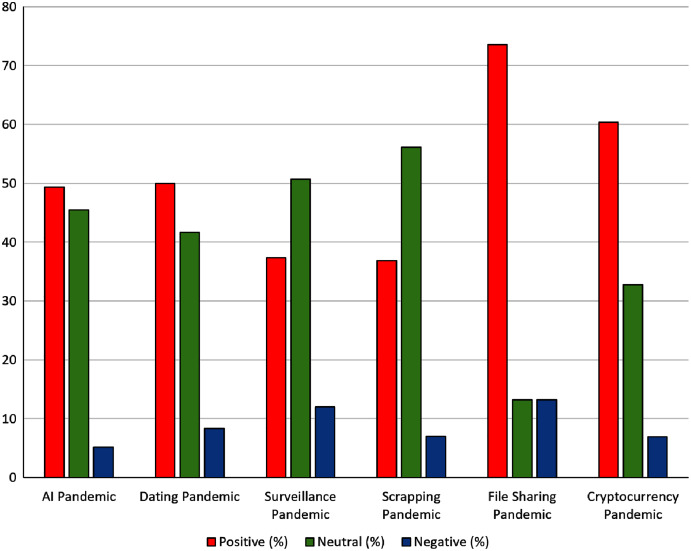
Comparison of sentiment analyses in social media among several IT strategy fields in pandemics.

- Identify keywords for the business: In the particular IT company, the most relevant fields of work were identified with the keywords scraping tools, artificial intelligence (AI), ML, surveillance, dating apps, emerging social networks, files sharing, emerging mobile apps, cryptocurrency trading, and investment.
- Find relation with pandemic keywords: The scraping tool identified the most relevant pandemic words associated with the aforementioned IT concepts with support of the Social Mention website. Besides the common word of Covid19, we also found interesting the word “recovered” as new emerging business strategies with focus on the infected people that were recovering.
- Perform sentiment analysis on social media: In the social media, the combination of AI and pandemic obtained a positive sentiment analysis, as Fig. 4 shows. Moreover, we performed a comparison of the most relevant combinations, which is presented in Fig. 5.
- Identify most relevant monitoring websites: Some of the most relevant information was found in *nbcwashington.com* and *businessofapps.com*. A total of one website indicated what was expected by dating app users in this pandemic context, and this information was useful for designing this kind of apps. The other website provided useful information about revenues of certain apps in pandemics, to estimate which kind of app might be more successful in the changing context of pandemic. Like in the previous scenario, a ranked list of websites was obtained by scraping on Social Mention website, and the IT company owner selected the most relevant websites from the first items of the provided list.
- Generate scraping tool: Our proposed framework generated scraping tools for these websites and the combination of words. However, the generated scraping tools were manually adapted to obtain more detailed information like including the revenues.
- Automatic monitoring: In the automatic monitoring, we established a daily frequency of this analysis, so that the company could use this updated information every day.
- Visualize clustering map: The clustering map was visualized considering similarity and sentiment for each combination. The most relevant cluster of documents had a center with document similarity of 69.1% and a sentiment of 67.2%. After analyzing all the documents of this cluster, both “file sharing” and “Zoom” were the most frequent terms considering only the IT concepts and tools.
- Generate explanation with HAI: An example of generated explanation was “*In the learned model for business opportunities in pandemics, the most relevant input combination for estimating that you have negative emotions about vaccines is that you were thinking about AstraZeneca and clots*”. The IT expert observed an opportunity in an app that helped users to reply a questionnaire about their symptoms after being injected AstraZeneca vaccine to quickly indicate their probability of suffering clots.
- Collect feedback from users: The users indicated which web pages were relevant from the ones retrieved by the proposed tool, for conforming new business strategies.
- Supervised learning: The Multinomial NB, MLP, KNN and SVM models were updated with the users’ feedback for improving the retrieval of relevant elements.

Figure 5 presents the number of documents classified in the different sentiment categories for each combination of business term and pandemic term, providing more insight information than rather the average sentiment considered in the clustering map. For instance, it is not the same to have a concept that have most documents classified as neutral (like for example scraping in pandemics) or others with either many documents classified as either positive or negative (not any case in this example). “File sharing” and “cryptocurrency trading” raised the higher numbers of documents with positive emotions. More concretely, the combination of words with a highest percentage of documents with positive sentiments was “File Sharing” and “Pandemic”, with 73.5% of documents with positive emotions. This may reflect that pandemic situations implied a higher need of sharing files, and IT businesses may increase their chance of success if their products include more options related with file sharing. The combination of “cryptocurrency” and “pandemic” was the second most related one with positive emotions (*i.e.*, 60.3%). This helped the user to assess the possibility of integrating automatic cryptocurrency trade on their skills and services.

**Figure 6:**
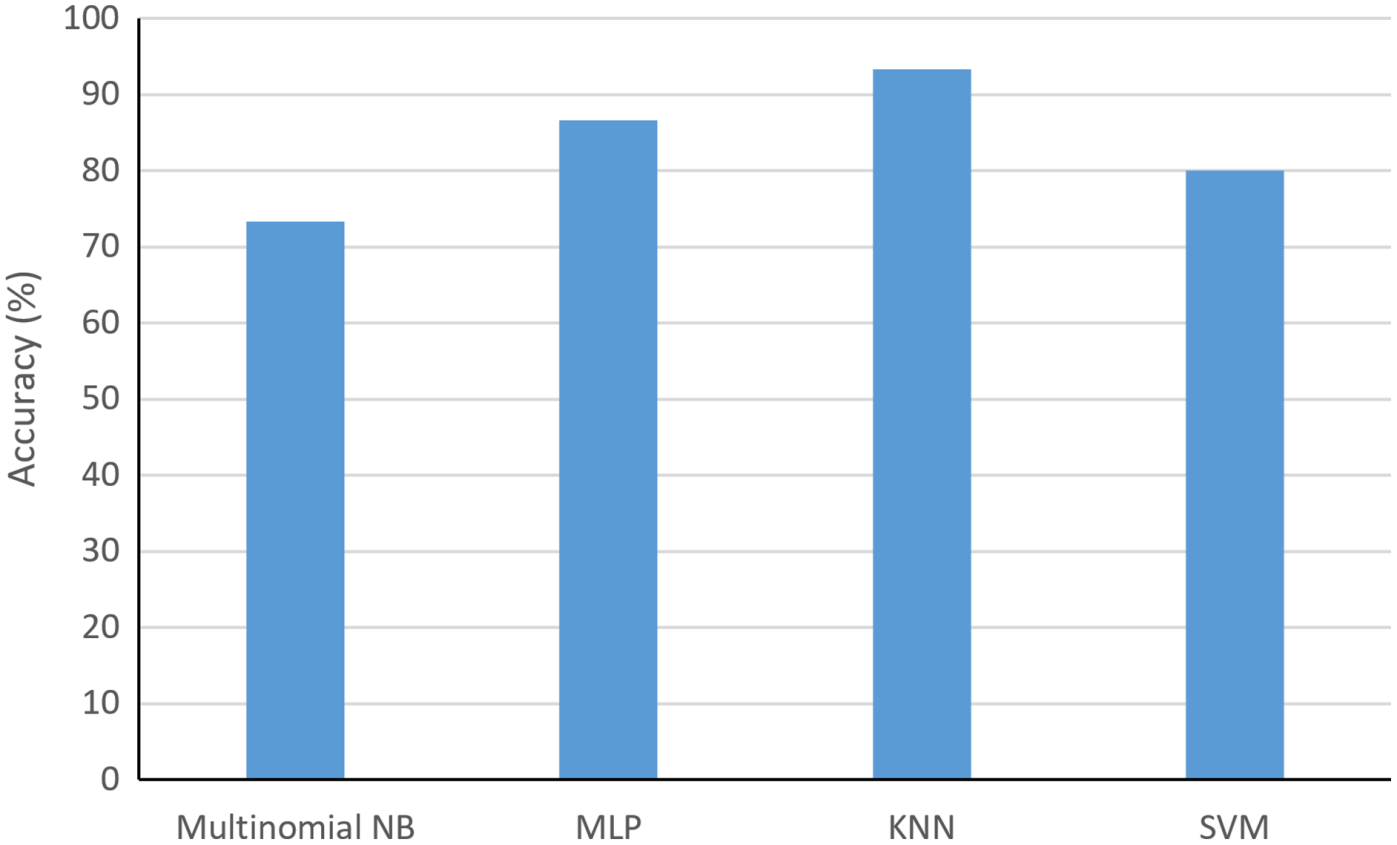
Comparison of accuracy when using different ML classifiers in the context of AI and pandemics.

In order to assess the efficacy of supervised learning, a user tagged an initial set of 11 documents retrieved from Social Mention with the terms of AI and pandemics, using the categories of ‘relevant’ documents and ‘other’ documents. These were used for the training. Then, 15 documents were automatically classified considering multinomial NB, MLP, KNN and SVM. The user also tagged these documents, so that the accuracy of the classification could be calculated. Figure 6 shows the accuracy for the different classifiers.

As one can observe, the highest accuracy was obtained with KNN with a value of 93.3%. The second highest accuracy was obtained with MLP with a value of 87.7%. On the contrary, the multinomial NB obtained the worst accuracy with a value of 73.3%.

**Figure 7:**
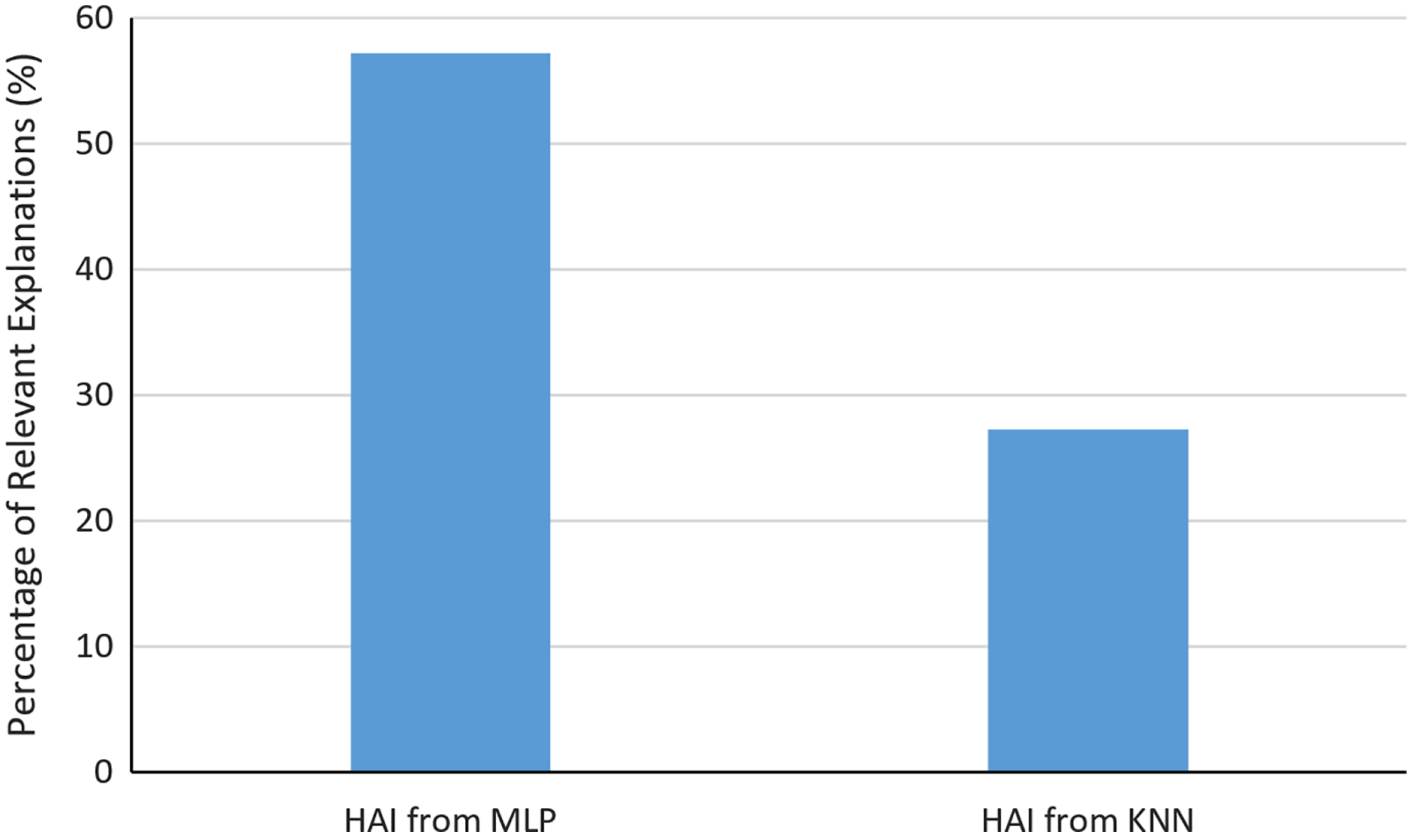
Percentage of relevant explanations automatically generated with HAI.

In order to evaluate the automatically generated explanations, we asked the users to determine which explanations were relevant for them for identifying business opportunities. In the analysis of seven explanations generated with HAI from explainable MLPs and 11 explanations generated with HAI from KNN, 57.1% of the generated explanations from MLP were found relevant, and 27.3% of the explanations from KNN were found relevant. Figure 7 shows the comparison of these percentages. One can observe that in general explainable MLPs provided a higher ratio of relevant explanations than the analyzed alternative.

## Conclusion and future work

This work has presented a novel approach for conforming a scraping tool that assisted business men in identifying which strategies were most appropriate for maintaining their companies profitable in pandemics, considering data from different sources through data fusion. It use d a supervised ML approach for estimating which online documents could be relevant for a given user. In this way, the system filtered the documents presented to users. The system automatically generate d explanations for providing ideas with HAI for identifying business opportunities.

As future work, we plan to improve the proposed tool by using more documents tagged by sentiments from more sources, so that the results are more representative. We also plan to automate the finding of customers by digging into social messages related with the most promising business strategies, enhancing the input data from more sources. We will also explore the application of other HAI techniques aiming at increasing the quality of explanations. We plan to propose HAI techniques in deep learning by identifying patterns in the layers of neurons and using statistical information from the input data, although this is widely known to be really challenging. We also plan to apply HAI over k-nearest neighbours algorithm through adaptation of the explanation from the most similar cases, since we believe this might be the most straightforward way of improving the user experience when reading the generated explanations.

## Supplemental Information

10.7717/peerj-cs.713/supp-1 Supplemental Information 1 Python programming code.

## Additional Information and Declarations

### Data Availability

The following information was supplied regarding data availability: All the programming code uses Python programming language and is available in the Supplemental File.
